# Influenza: temporal trends in mortality, hospitalization, and vaccination in the older adults population aged 60 and more years, Brazil, 2000–2023

**DOI:** 10.3389/fpubh.2025.1615503

**Published:** 2025-09-01

**Authors:** Erly Catarina Moura, Juan Jose Cortez-Escalante, Fabrício Vieira Cavalcante, Leonor Maria Pacheco Santos, Wallace Enrico Boaventura Gonçalves Dos Santos

**Affiliations:** ^1^Faculty of Health Sciences, University of Brasília, Brasília, Brazil; ^2^Department of Collective Health, Brasília, Brazil; ^3^Pan American Health Organization, Bogotá, Colombia

**Keywords:** influenza, mortality, hospitalization, vaccine, older adults population

## Abstract

**Objective:**

To evaluate the temporal evolution of mortality rates, hospitalization, and vaccination coverage for influenza among the population ≥ 60 years of age in Brazil from 2000 to 2023. Methods A descriptive study with secondary and anonymous data from the Ministry of Health was collected year by year to calculate the rates. Data on the composition of the vaccines were also obtained.

**Results:**

The data pointed to: (1) an increase in deaths from influenza, especially during the COVID-19 pandemic, but a decrease in 2023 with the registration of 2.5 deaths per 100 thousand inhabitants; (2) reduction in hospitalizations for influenza, but increase during the Covid-19 pandemic and decrease in 2023, with a record of 22 hospitalizations per 100 thousand inhabitants; (3) linear growth in vaccination coverage, reaching 99.4% in 2019, exceeding 100% in 2020 and a sharp drop in subsequent years, reaching the lowest value in the series (63.3%) in 2023; and (4) formulation of vaccines containing three different strains in all campaigns.

**Conclusion:**

The impact of Covid-19 on mortality and hospitalization rates from 2020 beyond is evident. The role of the federal government in controlling pandemics and the importance of vaccination, among other measures, are highlighted. Currently, the challenge is to increase immunization against influenza, a disease with low mortality and hospitalization rates for the population of this study, but with high transmissibility in the general population, with an impact not only on health, but also on social and economic wealth.

## Introduction

The World Health Organization (WHO) estimates that one billion people are affected by influenza each year (1), a disease responsible for major outbreaks, epidemics, and pandemics worldwide. Notable events (2) include the Spanish flu in 1918–1919, the Asian flu (subtype H2N2) in 1957–1958, the Hong Kong flu (subtype H3N2) in 1968, and the swine flu (subtype H3N2) in 2009, besides a suspected outbreak in the year 412 BC. Influenza is a seasonal acute respiratory infection, transmissible from person to person, but there is also transmission from animals, such as chickens, cows, and pigs, being H3N2 the precursor of the subtype H1N1 in humans (1).

Typically, the disease manifests one to 4 days after infection and is mainly characterized by fever, cough, headache, muscle pain, malaise, sore throat, and runny nose (1, 3). In addition to health problems, influenza causes difficulties from a social and economic point of view, due to its high virulence (1, 4), which created the need for a large-scale preventive measures, leading to the development of vaccines since 1930 (5).

There are four types of viruses (A, B, C, and D) and more than 30,000 subtypes/lineages. The first three types infect humans, while type D has only been identified in animals so far. Types A and B are responsible for pandemics in humans, while C is more contained and less aggressive (1, 6).

At the end of the last century, type A viruses, subtypes H1N1 (swine origin) and H5N1 (avian origin), were widely disseminated on the planet (2), requiring the Brazilian Ministry of Agriculture, Livestock and Supply to implement a surveillance program for the virus in birds (7). This action, together with the influenza surveillance measures implemented by the Ministry of Health in 2000 (8), characterized mainly by the implementation of sentinel units and the notification protocol, were fundamental in the rapid control of the H1N1 pandemic in Brazil. These resolutions probably had an impact on reducing hospitalization rates and rapidly reducing mortality rates, a successful example of the One Health strategy, an integrative approach that recognizes the interconnectedness and interdependence between humans, animals, and the environment (9).

In Brazil, influenza vaccination began in 1999, for those aged 65 years and over, and was expanded to 60 years or older in 2000 (10) and to other priority population groups. Since then, vaccination campaigns have become annual and follow recommendations based on circulating viruses; thus, the vaccine composition changes each year and cannot be stockpiled (10).

The older adults are very sensitive to contracting the infection, with high rates of hospitalization and mortality, and are considered a priority group in public health, with the recommendation of annual mass vaccination campaigns, according to the circulating viral subtypes (1).

The Brazilian Ministry of Health reorganized its surveillance infrastructure in 2003 (11, 12) bringing together surveillance, prevention and control of communicable diseases, previously dispersed, such as the National Immunization Program created in 1973 (13), and the National Center of Epidemiology. The National Immunization Program was expanded, granting, free of charge, annual vaccine availability (13). However, challenges remain to ensure high coverage and to reduce regional disparities in healthcare access. Additionally, new surveillance systems created, including that for monitoring severe acute respiratory infections, “*SIVEP-gripe”* (14) plays a crucial role in responding to influenza outbreaks among the older adults; it also proved instrumental in the early stages of the Covid-19 pandemic (15). These changes were important for public health in general, and especially influenza control, mainly among the older adults. Broadening the approach, in 2024 Brazil established the Interinstitutional Technical Committee of One Health, involving various ministries and institutions to prepare and revise the National Action Plan (16).

This article focuses on influenza in Brazil in the 21^st^ century among individuals aged 60 years or older, aiming to assess trends in mortality, hospitalization, and vaccination.

## Methods

A descriptive study of time series was carried out with secondary and anonymous data, involving all residents aged 60 years or older. Official data were obtained directly from the Ministry of Health, stratified by year.

Data from the Mortality Information System (17) were used to identify deaths from influenza when the underlying cause was coded as J09 (Influenza A - H1N1), J10 (influenza due to another viruses), or J11 (influenza due to unidentified viruses), according to the International Classification of Diseases and Related Health Problems (ICD-10) (18).

All hospital admissions were obtained from the Hospital Morbidity of the Brazilian Unified Health System (SUS) (19), with influenza-related hospitalizations identified using the same ICD-10 codes (20).

Vaccination coverage rates for influenza (expressed as percentages) were obtained from the Immunization Program Evaluation System (2000–2010) (21) and the National Immunization Program Information System (2011–2022) (21). Data for 2023 were obtained from the Ministry of Health’s official influenza campaign portal (22).

Rates of all-cause mortality (per 100,000 inhabitants), influenza specific mortality (per 100,000 inhabitants), all-cause hospitalization (per 100,000 inhabitants), influenza-specific hospitalization (per 100,000 inhabitants), and influenza vaccination coverage (per 100 inhabitants) were calculated using population estimates based on the most recent national census data (23).

This study does not require the approval of the Ethics Committee for Research with Human Beings, as only secondary and anonymous data were used (24).

## Results

**Figure 1:**
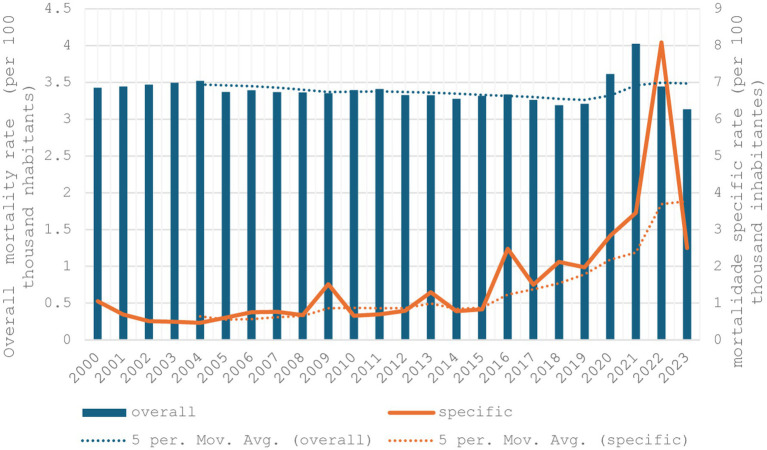
Overall mortality rate (per 100 thousand inhabitants) and influenza-specific mortality rate (per 100 thousand inhabitants) in individuals aged 60 years and over, Brazil 2000 to 2023.

Since 2000, all-cause mortality rates among individuals aged 60 years and over in Brazil have ranged between 2.5 and 3.5 per 100,000 inhabitants. During the Covid-19 pandemic (2020–2021), these rates rose to 4.0 per 100,000 inhabitants, before decreasing to just above 3.0 per 100,000 in 2023 (Figure 1). Influenza-specific mortality showed a slight decline over the preceding 3 years (2000–2003), followed by an upward trend beginning in 2007. Notable peaks occurred in 2009, 2016, and 2022, reaching over 8 deaths per 100,000 inhabitants in 2022.

**Figure 2:**
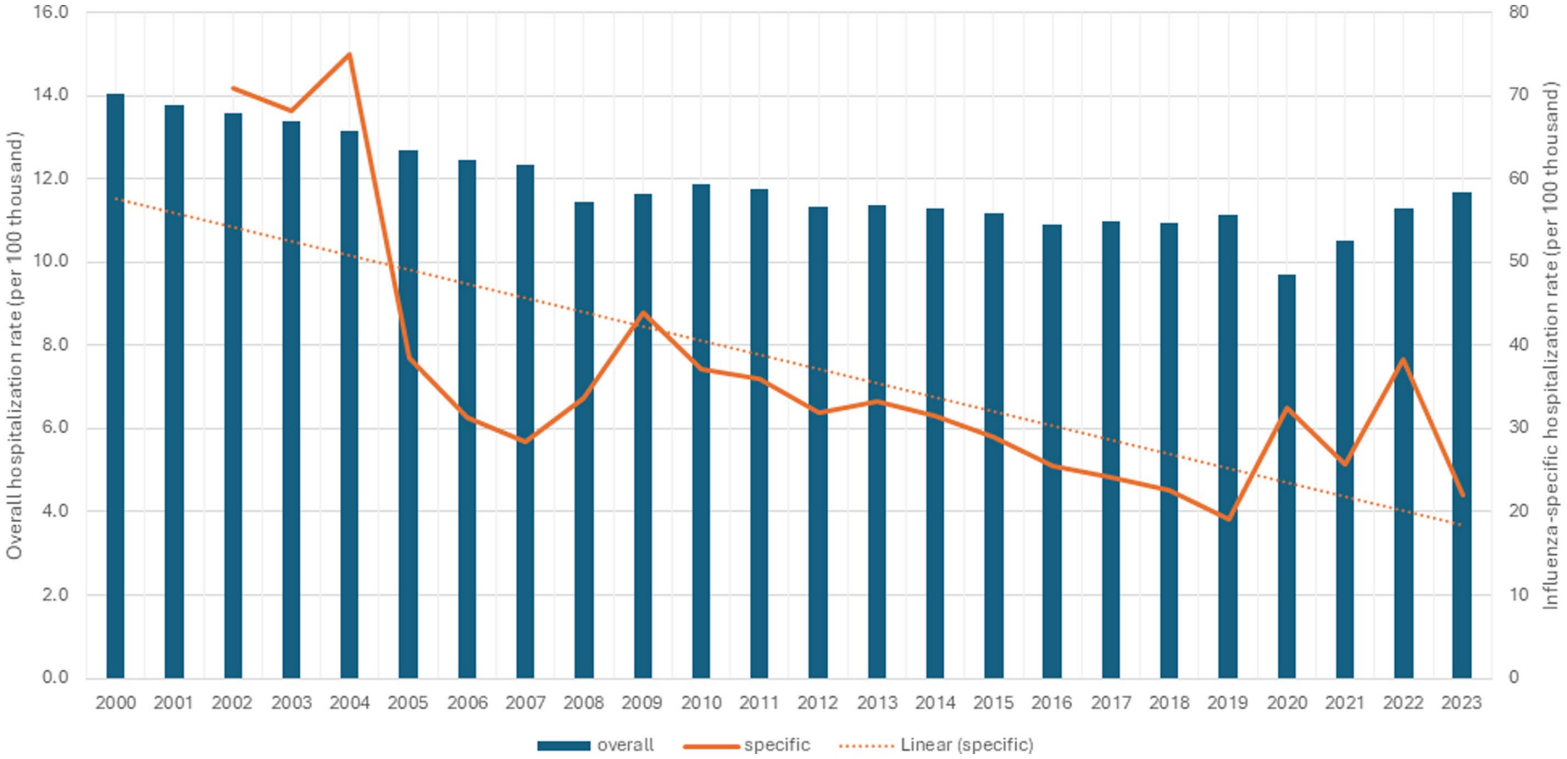
Overall hospitalization rate (per 100 thousand inhabitants) and influenza-specific hospitalization rate (per 100 thousand inhabitants) in individuals aged 60 years and over, Brazil, 2000 to 2023.

Figure 2 displays trends in all-cause and influenza-specific hospitalization rates. Overall, all-cause hospitalizations declined from 14 per 100,000 inhabitants in 2000 to 9.7 in 2020, with an increase observed over the subsequent 3 years. In 2023, the all-cause hospitalization rate reached 11.7 per 100,000 inhabitants. Mandatory reporting of influenza-related hospitalizations began in 2002 in the “*SIVEP-gripe*” system. Initially, rates rose to 70 per 100,000 inhabitants, followed by a marked decline, reaching approximately 19 per 100,000 inhabitants in 2019. Peaks were observed in 2009 (43.7), 2020 (32.9), and 2022 (37.6), with a subsequent decrease in 2023.

**Figure 3:**
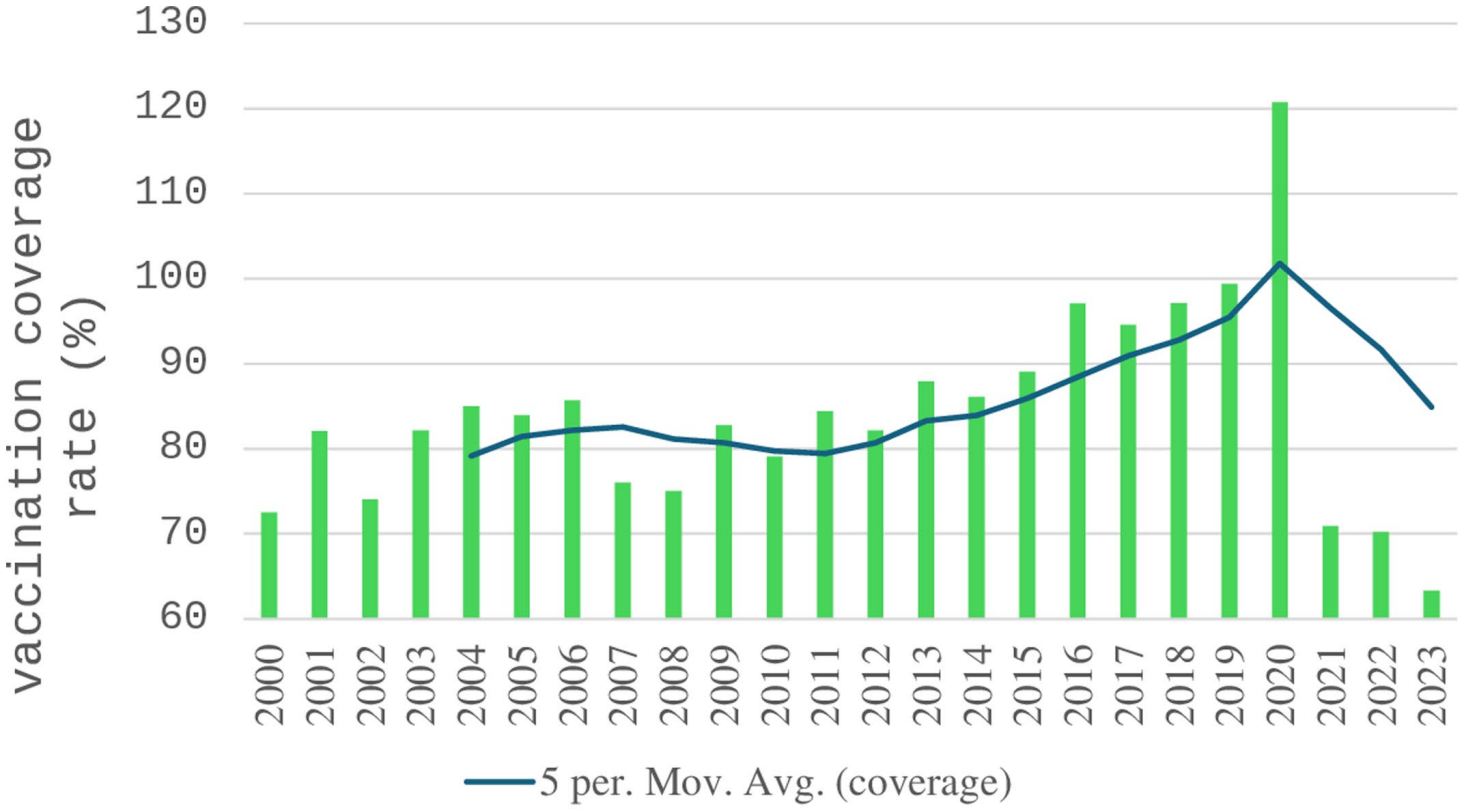
Influenza vaccination coverage rate (%) in individuals aged 60 years and over, Brazil, 2000 to 2023.

Influenza vaccination coverage among individuals aged 60 years and over was 72.5% in the first year of vaccination (2000), showing a general increasing trend in the period evaluated (Figure 3). Average coverage was approximately 80% from 2000 to 2008, 85% from 2009 to 2015, and 97% between 2016 to 2019. Coverage exceeded 120% in 2020, indicating people with more than one dose, followed by a sharp decline, reaching a low of 63.3% in 2023—the lowest rate in the entire series.

Table 1 presents the composition of influenza vaccines used in Brazil between 2000 and 2023. All formulations included three strains: two type A (H1N1 and H3N2) and one type B. Over the study period, 7 H1N1 strains, 16 H3N2 strains and 13 distinct B strains were used.

**Table 1:** Formulation of influenza vaccines according to strains recommended by the Ministry of Health and year of vaccination campaigns, Brazil, 2000 to 2023.

Strains			
Year	Type B	Type A (H1N1)	Type A (H3N2)
2000	Beijing/184/93	New Caledoni20/99	Moscow/10/99
2001	Sichuan/379/99		
2002			
2003	Hong Kong/330/2001		
2004			Fujian/411/2002
2005	Shanghai/361/2002		Wellington/1/2004
2006	Malaysi2506/2004		Californi7/2004
2007			Wisconsin/67/2005
2008	Florid4/2006	Solomon Islands/3/2006	Brisbane/10/2007
2009		Brisbane/59/2007	
2010	Brisbane/60/2008	Californi7/2009	Perth/16/2009
2011			
2012			
2013	Wisconsin/1/2010		Victori361/2011
2014	Massachusetts/2/2012		Texas/50/2012
2015	Phuket/3073/2013		Switzerland/9715293/2013
2016	Brisbane/60/2008		Hong Kong/4801/2014
2017		Michigan/45/2015	
2018	Phuket/3073/2013		Singapore/INFIMH-16-0019/2016
2019	Colorado/06/2017-like virus		Switzerland/8060/2017
2020	Washington/02/2019	Brisbane/02/2018	South Australi34/2019
2021		Victori2570/2019	Hong Kong/2671/2019
2022	Austri1359417/2021		Darwin/9/2021
2023		Sydney/5/2021	

Table 2 condenses all the data from this study.

**Table 2:** Influenza data (mortality, hospitalization and vaccination coverage) in individuals aged 60 years and over, Brazil, 2000 to 2023.

Year	Mortality (rate per 100,000)	Hospitalization (rate per 100,000)	Vaccination coverage (%)
2000	1.05	2.36	72.5
2001	0.69	1.42	82.1
2002	0.51	70.89	74.1
2003	0.49	68.15	82.1
2004	0.46	75.06	85,0
2005	0.61	38.59	83.9
2006	0.75	31.34	85.7
2007	0.77	28.36	76.0
2008	0.67	33.58	75.1
2009	1.52	43.91	82.8
2010	0.66	37.16	79.1
2011	0.69	35.92	84.4
2012	0.79	31.90	82.1
2013	1.29	33.37	88.0
2014	0.78	31.55	86.1
2015	0.83	29.04	89.1
2016	2.48	25.51	97.1
2017	1.50	24.15	94.6
2018	2.12	22.63	97.2
2019	1.98	19.13	99.4
2020	2.84	32.58	120.7
2021	3.46	25.80	70.9
2022	8.09	38.32	70.2
2023	2.50	22.07	63.3

## Discussion

This study showed that influenza-specific mortality among older adults people aged 60 years and older in Brazil is relatively low compared to all-cause mortality, according to 5 years moving average for linear regression, although notable peaks were observed in 2009 and 2022, in line with WHO global estimates (1). Both, in 2009 when the WHO declared the Public Health Emergency of International Concern for H1N1 (2), and in 2022 during the Covid-19 epidemic, which may partly reflect Covid-19-related mortality misclassified as influenza, given that older adults represent the age group at highest risk for severe outcomes and death from SARS-CoV-2 infection.

The peak in influenza-specific mortality may include false-positive reports due to limited availability of confirmatory molecular testing, which hampered differential diagnosis between influenza and Covid-19, meanwhile, both for H1N1 (25), and Covid-19 (26), the ≥ 60-year-old population were considered as a risk group. It is possible that Covid-19 deaths were mistaken for viral influenza infection. In 2023, with the possibility of the correct laboratory identification of the coronavirus, there is a drop in the influenza mortality rate.

Regarding hospitalization rates, it is remarkable that the registration of influenza as a cause of hospitalization had a great advance after 2001, following the first vaccination campaign in 2000. Possibly sensitized not only by the campaign itself, but also by the comprehensive mass media dissemination, health services likely became more attentive to properly coding influenza-related hospitalizations, especially after mandatory reporting began. After the fifth annual campaign, there was a marked reduction in hospitalizations due to influenza between 2004 and 2005. Vaccination is the main responsible to limit the burden of the disease and consequently reduce hospitalization in the ≥60-year-old population (27). As with mortality rates, the impact of the Covid-19 pandemic on hospital admissions is observed, with a slight increase in 2020, possibly due to greater surveillance, individual and health services, for cases of respiratory infection.

The reduction in reported hospitalization reflects the implementation of annual vaccination campaigns and the mandatory notification of influenza, aiming at the development of strategies to mitigate cases (1). The Brazilian Universal Health System’s regionalization defines the flow of patients according to the complexity level of health services in the territory (geographic regions and health macro-regions), looking optimizer municipalities installed capacity and people transport availability (28, 29), essential in the prevention of serious outcomes, especially among the older adults population. Disparities in health infrastructure across regions of Brazil may have influenced hospitalization rates (30), because differences in installed capacity (equipment, hospital beds, and health professionals), particularly low in the North and Northeast regions of the country (31).

Otherwise, the Covid-19 pandemic was accompanied by a wave of infodemic, reaching various groups in the Brazilian society, stressing disease magnitude and severity (32). Numerous conspiracy theories emerged during the Covid-19 pandemic period, for instance: anti-vaccination demonstrations (33), government discourses encouraging the use of drugs proven to be ineffective against Covid-19 (34), and inspiring agglomeration of people.

In both indicators, mortality and hospitalization rates, the peak in 2009 is notable, which can be explained by the H1N1 (35) pandemic, characterized by human-to-human transmission and rapid international spread facilitated by high global mobility, leading the WHO to declare a state of emergency (36). In 2013 and 2016, the peaks in influenza mortality were also due to the H1N1 virus.

Comparison with data from other studies needs to be cautious, because of the possible difference in method and because of the various characteristics of the population evaluated and the study period. In the southern region of Brazil, for example, Freitas et al. (37) estimated that influenza-related deaths were 1.8 per 100,000 due to excess mortality in 2000 and 4.5 in 2007. However, the present study points to a reduction from 1.05 to 0.77 in the same period for the entire country.

In Germany (38), modeling by excess deaths pointed to an influenza mortality rate of 35.35 per 100,000 and hospitalization of 117.26 per 100,000 for the population aged 60 and over, on average, in the period from 1996 to 2018. The present study points out, for Brazil, an average mortality rate equal to 0.98 per 100 thousand in the period from 2000 to 2018 and an average hospitalization rate equal to 38.89 per 100 thousand in the period from 2002 to 2018.

Data from the United States (39) show, for 2023–2024, in people aged 65 and over, a mortality rate equal to 32.1 per 100 thousand inhabitants and 400.5 hospitalization. Despite the age difference, the mortality rate is like that of Brazil for the year 2022, but the hospitalization rate is almost 11 times higher, which can be explained by the difference in age structure and health care.

In Portugal (40), the mean annual hospitalization rate, using the same ICD-10 (20) classification used in this study, for primary or secondary diagnosis, was 199.6 per 100 thousand for people aged 65 years and over, in the period between 2010 and 2018, while our study presented an average of 30.14, only for primary diagnosis, for people aged 60 years or older, in the same period. A study close to that of Portugal indicated a hospitalization rate of 335.3 per 100 thousand in Spain (41).

About vaccination, there is an increase in coverage over time, especially in 2020, the year of the Covid-19 eruption, with coverage above 100%, followed by a drastic decline, possibly influenced by reduced public trust, vaccine hesitancy, and limited governmental promotion during the Covid-19 pandemic. The impact of immunization on the control of mortality and hospitalization is evident, especially since 2009. Then, coverage rates above 100% may reflect discrepancies in population estimates or vaccination of individuals outside the target age group.

In the United States, vaccination coverage in individuals aged 65 years and over in 2011–12 was equal to 69.4% (42), increasing to 75.2 in 2020–2021 (43), while in Brazil, the average, respectively for the same periods, was higher than 83% and almost 96%. Foppa et al. (44), evaluating excess influenza mortality in the United States, estimated an 88.9% reduction in deaths due to vaccination in people aged 65 years and over between 2005/2006 and 2013/2014.

Gharpure et al. (45), in a case-control study carried out in 2023, involving eight countries, including Brazil, found that the influenza vaccine is effective in reducing hospitalization, but Brazil had efficacy below 40% for the strains evaluated. This reduced efficacy may be attributed to a mismatch between vaccine strains and circulating viruses, as well as potential delays in campaign execution. It is important to note that the vaccine formulation in Brazil since 2000 has three different strains: one type B and two type A.

In this sense, Lejarazu and Tamames (46) advocate the inclusion of two strains in the formulation of the influenza vaccine, namely *Yamagata* and *Victoria*, instead of just one. This is reinforced by the high rates of mutations and antigenic variation, pointing to the need for annual reformulation of vaccines aiming to increase effectiveness against the circulating virus (47).

Corroborating the above position, Trigo et al. (48) reported the advantages of the Quadrivalent vaccine, including one more strain of the type B in the formulation of the trivalent vaccine. They also show the enhancement of the cost-effectiveness relationship in reducing mortality and hospitalization rates and in the impact of the disease from a health, social and economic point of view.

Following the coronavirus (COVID-19) pandemic, there have been changes in the global circulation of influenza viruses, leading to the possible elimination of the type B (*Yamagata*) (49), which has not been conclusively detected since April 2020. With the disappearance of this lineage, the use of the quadrivalent vaccine is no longer justified. Although it has become common in Europe, but not in Brazil.

Andrew et al. (50), considering the reduction in immune function and inflammatory responses with aging, advocate not only increasing vaccination coverage in the older adults, but also improving the efficacy of vaccines, pointing out the need to reformulate them, a position also defended by Uyeki et al. (51).

In 2010, in response to the emergency alert decreed by the WHO, Brazil, in a collective effort of the government, vaccinated 42% of the total population against influenza, a value much higher than that observed in other countries such as the United States: 26%, Mexico: 24%, Switzerland: 17%, Argentina: 13%, France: 8% and Germany: 6% (39). This action, combined with the vaccine formulation containing three different types of strains, made it possible to control the disease in Brazil. However, recent data show very low vaccination coverage in the country, the appearance of new strains in the region of the Americas (52), and the early onset of seasonal outbreaks (53, 54).

The influenza epidemic experiences of countries such as Germany, Portugal, and Spain highlighted the importance of robust syndromic surveillance systems, decentralization of access to laboratory diagnosis, and health promotion strategies that motivate timely search (38, 40). In this regard, Brazil increased investments in laboratory diagnosis through the Growth Acceleration Program in 2023 (55), and the signing of a proposal agreement established by the WHO with more than 120 countries to face future pandemics (56). Brazil is a model in terms of vaccination campaigns (39), providing annual vaccination based on circulating viral composition, aligned with the strengthening of sentinel units (8).

The vaccine has been considered the best protection against influenza, but coverage is still low, including among health professionals (57, 58). However, studies point to the lack of knowledge and timid public policies as the biggest obstacles (59). A possible explanation is the population’s lack of knowledge about the possible adverse effects of the vaccine and the need for health units to notify them. Monteiro et al. (60) described the adverse effect surveillance system as simple, but with low sensitivity and overestimation of severe effects.

Gil-de-Miguel et al. (54) argue that the impact of influenza on society is underestimated. In a review study carried out in Spain and Portugal, they point out the need to reduce the impact of the disease, particularly in the older adults, and the presence of comorbidities increases the risk regardless of age.

In addition to underreporting, Costa and Merchan-Hamann (2) warned in 2016 of the importance and need for an articulated Brazil government response, involving health system managers at the three levels of government, likewise to scientific institutions in the area. The federal government is the main responsible for combating scientific denialism and vaccine hesitancy, encouraging studies to reformulate the flu vaccine, in addition to the challenge of expanding vaccination coverage and articulation between the different levels of government in the fight against influenza.

The influenza response in Brazil, as recommended by the WHO (1), is characterized by continuous monitoring, including virus identification, vaccine development, hospitalization, and death surveillance. In Brazil, the population aged 60 years and over grew from 8.5% of the country’s total population in 2000 to 15.6% in 2023. With the demographic transition and changes in population pyramids, this situation will become an immense challenge to face the Unified Health System in the coming years. This increase requires differentiated attention to the older adults population with specific actions for health promotion and protection, risk prevention and individual care. The recent drop in influenza vaccine coverage from 2021 onwards is worrying, alerting the new Brazilian health authorities (who took office in 2023) to the need for emergency actions in this matter.

Brazil’s prior experience with avian influenza surveillance and coordinated responses during the H1N1 pandemic exemplify the effectiveness of its integrative “One Health” approach. To sustain and expand these efforts, Brazil should prioritize enhanced multisectoral collaboration among public health, veterinary, and environmental sectors, supported by integrated surveillance systems for early detection and response to emerging zoonotic diseases. Strengthening environmental monitoring to address ecological changes driven by climate change and biodiversity loss, which heighten zoonotic risks, is also essential. Additionally, policies promoting continuous training and community engagement in “One Health” principles can further reinforce national preparedness. These integrated strategies will enable Brazil to proactively manage emerging zoonotic threats, as well as to antimicrobials in an increasingly vulnerable global environment.

This study presents some limitations: data about “influenza-specific mortality” and “influenza-specific hospitalization” may include false- positives due to limited availability of confirmatory tests; lack of a direct causal relationship between exposure and outcome, making it is not possible to accurately infer the causal factors associated with influenza cases and deaths in the population aged 60 years and over.

Specifically, during pandemic periods such as the 2009 H1N1 outbreak and the Covid-19 pandemic years of 2020 to 2022, the availability of supplies and access to molecular diagnostic tests for influenza confirmation were severely limited. This situation inevitably affected the precision of case identification, increasing the likelihood of false-positive reports or misclassification of cases, particularly between influenza and Covid-19 infections, which share similar clinical presentations. This diagnostic uncertainty is a recognized limitation and highlights the urgent need for strengthening diagnostic infrastructure and laboratory capacity in public health surveillance systems.

Improvements, like incorporating confidence intervals, sensitivity analyses, and multivariate approaches, are possible for future. Research efforts will enable a more robust and comprehensive understanding of the relationship between influenza vaccination and health outcomes in the older adults population in Brazil, ultimately contributing to more effective public health policies and interventions.

Furthermore, the dataset utilized - Hospital Information System of the Brazilian Unified Health System - is an administrative database primarily designed for billing and management purposes. Unlike electronic health records, Hospital Information System lacks comprehensive clinical details such as patient comorbidities, vaccination history, laboratory results, and other relevant individual-level data that would enable more nuanced analyses. This limitation constrains the depth of the investigation possible and precludes the ability to perform more sophisticated multivariate analyses that adjust for confounding factors.

Consequently, the current study did not include statistical measures such as confidence intervals or sensitivity analyses, nor did it employ multivariate modeling techniques that could provide greater insight into the independent effects of vaccination on hospitalization and mortality outcomes. The inclusion of these analytical methods would significantly enhance the rigor and interpretability of the findings by better quantifying uncertainty and controlling for potential confounders.

Although, his study provides a comprehensive analysis of long-term influenza trends among older adults in Brazil, highlighting critical impacts of the Covid-19 pandemic on mortality, hospitalization, and vaccine uptake. The findings reinforce the urgent need for reinvigorated immunization policies, improved public communication strategies, and sustained investment in surveillance infrastructure.

## Data Availability

Publicly available datasets were analyzed in this study. This data can be found at: http://tabnet.datasus.gov.br/cgi/deftohtm.exe?sih/cnv/nruf.def.
